# 667 Increased Access to Burn Therapy Services in Burn Urgent Care Clinic

**DOI:** 10.1093/jbcr/iraf019.296

**Published:** 2025-04-01

**Authors:** Rachel Szalony, Lindsey Harris, Velmaris Rodriguez-Ojeda

**Affiliations:** Parkland Health; Parkland Health; Parkland Health

## Abstract

**Introduction:**

Burn therapy is considered a key service throughout the continuum of care for burn survivors to promote function throughout the healing process and prevent functional complications from development of scarring and contractures as burn wounds heal. At our burn center, patients with unclosed burn or reconstructive wounds are seen on a weekly basis at an outpatient clinic staffed by a mid-level provider, a nurse, and a wound care tech. Access to therapy services in this clinic has been limited to referral to the outpatient therapy center in a separate building pending appointment availability or, in rare cases, an inpatient therapist leaving acute burn patient care to provide therapy services. The aim of this quality improvement project is to increase accessibility to and availability of burn therapy services overall and specifically for pediatric patients, including generation of outpatient therapy referrals, for burn survivors seen at a burn outpatient clinic.

**Methods:**

Three PDSA (plan, do, study, act) models were developed for increasing overall access to therapy care in the outpatient burn clinic, increasing pediatric access to therapy care in the outpatient burn clinic, and ensuring high service quality. Baseline scores from prior year were gathered, followed by education of clinic and acute care therapy staff regarding increased therapy presence and availability of therapy services in the clinic. Outpatient charting and billing education was provided for acute care therapy staff. Data was gathered for a 9-month period after program was initiated.

**Results:**

In the calendar year prior to program initiation, 9 total patients were seen by therapy including 1 pediatric patient. After program implementation, an average of 70.5 visits per month were provided to burn clinic patients and an average of 6.75 referrals per month for outpatient therapy outside of the clinic were generated. Of those average visits per month, 31.8 were pediatric patients with 2.4 pediatric patients per month referred for further outpatient therapy. Of 84 patient surveys collected regarding quality of therapy services, 98.8% of patients rated therapy services as good or greater.

**Conclusions:**

Increasing access to burn therapy services along the continuum ensures that burn survivors receive the care they need throughout their healing journey. This quality improvement program increased access to therapy services in an area of frequent contact with survivors in need of therapy services.

**Applicability of Research to Practice:**

With strategic assessment and education, burn centers can increase access to burn therapy services.

**Funding for the Study:**

N/A

